# Biogenesis of the Trypanosome Endo-Exocytotic Organelle Is Cytoskeleton Mediated

**DOI:** 10.1371/journal.pbio.0060105

**Published:** 2008-05-06

**Authors:** Mélanie Bonhivers, Sophie Nowacki, Nicolas Landrein, Derrick R Robinson

**Affiliations:** Microbiologie Cellulaire et Moléculaire et Pathogénicité (MCMP), UMR-CNRS 5234, University Bordeaux 2, Bordeaux, Cedex France; University of Oxford, United Kingdom

## Abstract

Trypanosoma brucei is a protozoan parasite that is used as a model organism to study such biological phenomena as gene expression, protein trafficking, and cytoskeletal biogenesis. In T. brucei, endocytosis and exocytosis occur exclusively through a sequestered organelle called the flagellar pocket (FP), an invagination of the pellicular membrane. The pocket is the sole site for specific receptors thus maintaining them inaccessible to components of the innate immune system of the mammalian host. The FP is also responsible for the sorting of protective parasite glycoproteins targeted to, or recycling from, the pellicular membrane, and for the removal of host antibodies from the cell surface. Here, we describe the first characterisation of a flagellar pocket cytoskeletal protein, BILBO1. BILBO1 functions to form a cytoskeleton framework upon which the FP is made and which is also required and essential for FP biogenesis and cell survival. Remarkably, RNA interference (RNAi)-mediated ablation of BILBO1 in insect procyclic-form parasites prevents FP biogenesis and induces vesicle accumulation, Golgi swelling, the aberrant repositioning of the new flagellum, and cell death. Cultured bloodstream-form parasites are also nonviable when subjected to BILBO1 RNAi. These results provide the first molecular evidence for cytoskeletally mediated FP biogenesis.

## Author Summary

Trypanosomes are ubiquitous unicellular parasites that infect humans, animals, insects, and plants. African, Asian, and some South American trypanosomes have evolved the amazing ability to change their surface coat proteins, an essential strategy for their survival. The surface coat proteins are recycled and targeted to the surface of the parasite via an endocytic and exocytotic organelle called the flagellar pocket, which is sequestered in the trypanosome cell's cytoplasm. The flagellar pocket is also used to remove host-derived antibodies that are bound to the surface of the parasite, making this organelle critical for the parasite's evasion of the host immune system. We describe a novel protein, “BILBO1,” which was identified from the insect-form parasite of the African trypanosome *Trypanosoma brucei.* We show that BILBO1 is part of a ring or horseshoe-like cytoskeletal structure that is located in a region of the flagellar pocket called the collar. When BILBO1 transcripts were knocked down with inducible RNA interference, trypanosome cells became arrested in a post-mitotic cell-cycle stage. Induced cells lost the normal flagellum-to-cell-body attachment, were unable to regulate endocytosis and exocytosis, and most importantly, were unable to construct a new flagellar pocket. These results provide molecular evidence for the idea that flagellar pocket biogenesis is cytoskeletally mediated.

## Abstract 3

RNAi of the parasite protein BILBO1 prevents the biogenesis of the endocytic and exocytotic organelle in *Trypanosoma brucei*, kills the parasite, and reveals novel insights into how this pathogen organizes and uses one of its distinctive organelles.

## Introduction

Endocytosis and exocytosis in trypanosomes is performed by the flagellar pocket (FP), an important organelle that is sequestered within the cytoplasm of the posterior region of the cell. On the basis of its protein composition, the FP membrane is biochemically distinct from the flagellar or pellicular membranes [1–3] and is also required for the molecular trafficking and recycling of glycosylphosphatidylinositol (GPI)-anchored proteins such as procyclin and variable surface glycoproteins (VSG). Both procyclin and VSG are surface coat proteins that are trafficked and recycled from the cytoplasm via the FP to the cell surface, where they function in the survival strategies of the cell. The molecular processes involved in these trafficking events are complex and require clathrin, actin, and a number of important GTPase Rab proteins [4,5].

An additional important feature of the FP is that it is physically linked to the cytoskeleton. This linkage can be observed in two areas of the pocket: (1) the axoneme of the flagellum traverses the FP prior to exiting the cytoplasm, and (2) the neck of the FP originates from a cytoskeletal structure that appears to be attached to the flagellum [6]. Similar to the sequence of events observed in kinetoplast segregation, the segregation of the new FP is precisely temporally and spatially coordinated, which implies that this process maybe mediated by the flagellum [7]. The site of FP biogenesis may also be mediated by the flagellar axoneme, as is observed for the Golgi apparatus of T. brucei [8]. The existence of a FP–cytoskeleton linkage would therefore explain the exquisite precision in positioning and segregation of the FP; namely that, the FP is always located on the proximal, cytoplasmic portion of the flagellum axoneme, and FP segregation is tightly coordinated with the flagellum biogenesis and segregation cycle.

The formation of a new flagellum is tightly associated with the maturation and elongation of the probasal body that is associated with the old flagellum [6]. The new axoneme then traverses the luminal core of the FP and exits the cell at a constricted site called the flagellar pocket collar (FPC) [9,10]. Although a number of proteins have been characterised to be specific to the FP, most are not essential, and none function in FP biogenesis [11–17]. For example clathrin and the proteins needed for receptor-mediated endocytosis are sequestered to the FP but are never exposed to the cell surface [2]. To date, data on FP organisation have been extremely limited and mainly based on ultrastructural studies. No proteins of the FPC have been identified, which is surprising in that the FP has the important role of controlling the targeting of molecules to and from the cell surface to avoid the host immune system [18–22]. Furthermore, endocytosis via the FP is not only used for the trafficking of parasite-derived molecules, but is used in the clearance of host antibodies bound to the cell surface [22–26].

In this study, we describe the identification and characterisation of the first cytoskeletal flagellar pocket protein: BILBO1. BILBO1 is a component of a cytoskeletal framework that is essential for biogenesis of the FPC. RNA interference (RNAi) ablation of BILBO1 in cultured procyclic insect forms (PF) of T. brucei prevents FP biogenesis, thus disturbing endocytotic activity and inducing vesicle accumulation, Golgi swelling, gross repositioning of the new flagellum, and cell death. Furthermore, cultured bloodstream forms (BSF) are not viable when subjected to BILBO1 RNAi in vitro. BILBO1, therefore, provides an interface between the cytoskeleton and the endocytotic and exocytotic systems, and represents the first molecular component of the FPC to be identified.

## Results

### BILBO1 Is the First Component Identified from the Flagellar Pocket Collar

**Figure 1:**
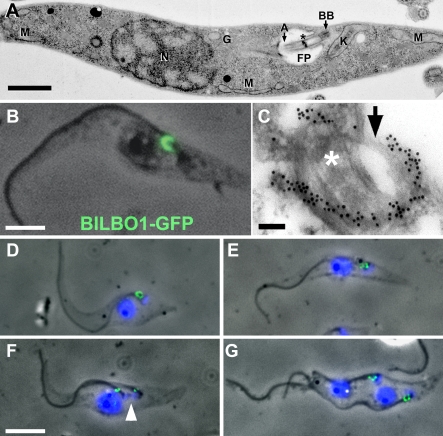
BILBO1 Localisation and FPC Biogenesis (A) A thin-section electron micrograph of a T. brucei PF cell. The image shows the structural characteristics of a 1K1N cell. Note; only trans-Golgi vesicles are observed, as the Golgi itself is not in the plane of the section. Asterisk (*) denotes the flagellum transition zone. Scale bar indicates 1 μm. A. axoneme; BB, basal body; G, Golgi; K, kinetoplast; M, mitochondria; N, nucleus. (B) Phase/fluorescence-micrograph of a procyclic cytoskeleton expressing BILBO1-eGFP. BILBO1-eGFP protein localises to the FPC. Scale bar indicates 2.5 μm. (C) Electron micrograph of a thin-sectioned WT cytoskeleton probed with anti-BILBO1 antiserum, followed by immunogold labelling. This illustrates the precise location of BILBO1 on the FPC. Asterisk (*) denotes the flagellum transition zone. Arrow denotes an unlabelled portion of the FPC. Scale bar indicates 100 nm. (D–G) Immunofluorescence of cytoskeletons probed with anti-BILBO1 antibody and counterstained with DAPI showing the FPC label and the duplication-segregation of the FPC during the cell cycle. Arrowhead in (F) denotes the kinetoplast, which has not completed S phase, and illustrates that the FPC is duplicated prior to kinetoplast S phase completion. Scale bar in (D–G) indicates 5 μm.

T. brucei has proven to be an excellent model for the study of cytoskeletal biogenesis [10,27]. T. brucei has several single-copy organelles, such as the mitochondrion, the kinetoplast (mitochondrial genome), a single Golgi apparatus, and a single FP. The endocytotic and exocytotic activity is limited to the posterior region of the cell via the FP. Figure 1A illustrates the morphology of a PF cell and shows the overall position of the FP within the cell. In PF cells, the FP is always closely associated with the flagellum, and both structures are always located in the posterior of the cell.

To attempt to characterise minor but essential proteins in the flagellum of T. brucei, salt-extracted flagellar proteins were separated by polyacrylamide gel electrophoresis (PAGE), and slices of the gel were used to immunise mice. Polyclonal serum obtained from these mice was then used to probe for novel proteins in immunofluorescence and western blotting studies. Proteins that appeared novel by immunofluorescence analysis were further investigated and eventually identified by mass spectrometry. From these studies, we identified a novel 67.3-kDa flagellar protein that we named BILBO1.

A single-copy gene, located on chromosome 11 of the T. brucei genome, encodes the BILBO1 protein. BLAST analysis, using parasite GeneDB databases, identified eight orthologs of *BILBO1*: one in *T. brucei gambiense*, one in T. congolense, two in T. cruzi (the South American trypanosome), one in T. vivax, one in Leishmania major, one in L. infantum, and one in L. braziliensis. With the exception of T. congolense, these genes have very similar locations with regard to their respective flanking genes, indicating that *BILBO1* gene synteny is preserved amongst these species. BLAST analysis of the genes of non-kinetoplastid organisms lacking a FP did not identify any other homologs to BILBO1. The primary and secondary structures of BILBO1 do not predict any localisation or cytoskeletal functions; however, this protein does possess two putative EF-hand calcium-binding motifs (amino acids [aa] 185–213 and 221–249), suggesting the existence of calcium binding sites and possible roles in regulation. The large C-terminus coiled-coil domain (aa 263–566) signifies a role in oligomerisation or protein–protein interactions.

In vivo overexpression of enhanced green fluorescent protein (eGFP)-tagged BILBO1 (Figure S1A) in PF cells localised the protein to the FPC (Figure 1B). In addition to the eGFP labelling experiments, we also raised antiserum to recombinant BILBO1 protein or peptides. Immunoelectron microscopy and immunofluorescence studies on PF cytoskeletons confirmed the eGFP-tagged BILBO1 localisation data (Figure 1C–1G). Identical immunofluorescence FPC–FP localisation was observed on BSF cytoskeletons (Figure S1B).

The anti-BILBO1 immunogold labelling observed in Figure 1C forms a horseshoe structure that is oriented around the emerging axoneme. No label is observed directly on the axoneme, suggesting that BILBO1 is present on the cell body side of the FPC as opposed to the flagellum side. However, when cytoskeletons are treated with 1 M NaCl, little or no BILBO1 protein is extracted, and the BILBO1 signal remains associated with the flagella preparation (Figure S1C). This indicates that, although BILBO1 is located on the cell side of the flagellum, the FPC and the BILBO1 protein both remain tightly linked to the flagellum. This observation is further supported by the presence of BILBO1 protein in the T. brucei flagella proteome [28].

### Biogenesis of the Flagellar Pocket Collar

In order to understand the biogenesis of the FPC, we examined cells in different cell-cycle stages labelled with the anti-BILBO1 antiserum. We observed that early in the kinetoplast S phase (as observed by kinetoplast DAPI staining), the old maternal FPC elongates and grows along its long principal axis, followed by a complete constriction of the short principal axis, thus forming two FPC structures (Figure 1E and 1F). During kinetoplast S phase, one of the FPC structures is moved towards the cell posterior along with new flagellum migration (Figure 1F and 1G). Figure 1F illustrates that division and segregation of the FPC occurs before kinetoplast S phase is completed. The new FPC appears to be segregated simultaneously with the new flagellum.

In wild-type (WT) cells, new flagellum segregation is accomplished by a subpellicular microtubule-mediated mechanism, which moves only the new flagellum towards the posterior end of the cell [7]. Since the FP is always physically linked to a flagellum, the simultaneous separation of the new flagellum and FP suggests that they may both be segregated by the same microtubule-mediated mechanism. Taken together, these data indicate that the mother FPC participates in daughter FPC biogenesis.

### BILBO1 Is Essential for Flagellar Pocket Biogenesis, Organelle Positioning, and Cytokinesis

**Figure 2:**
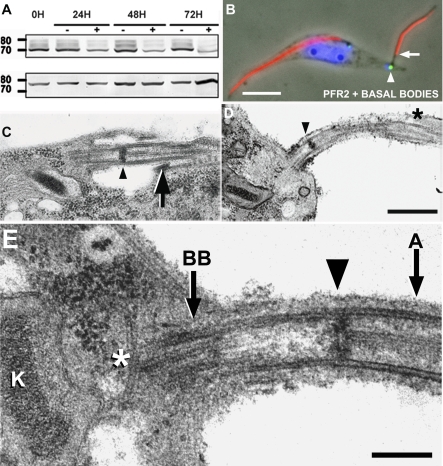
BILBO1 RNAi Prevents FP Biogenesis (A). Western blot of BILBO1 RNAi-induced cells (+) probed with anti-BILBO1 antibody (upper panel) or control antibody L8C4 (anti-PFR2), 0–72 h of BILBO1 RNAi induction. This blot shows that BILBO1 protein levels diminish over time but are still present after 72 h of RNAi induction. (B) A phase-DAPI-immunofluorescence micrograph of a BILBO1 RNAi-induced 2K2N cytoskeleton probed with anti-PFR2 (L8C4) and anti-basal bodies (BBA4) (36-h induction). Basal body and kinetoplast are indicated by the arrowhead; the flagellum and site of PFR initiation are indicated by the arrow. Scale bar indicates 5 μm. (C) A micrograph of a thin section of the FP of a noninduced PF cell illustrating the transition zone (arrowhead) and the FPC (arrow). (D) A micrograph of BILBO1 RNAi-induced cell (48 h) at the new flagellum region. Note loss of flagellum-to-cell body attachment, PFR (asterisk), transition zone location (arrowhead), and absence of a FP. Scale bar indicates 500 nm. (E) Thin-section micrograph of BILBO1 RNAi-induced (48 h) cell illustrating the proximal end of the new flagellum and the absence of a FP. A portion of the basal body (BB) is located in the cell, whereas the transition zone (arrowhead) is external to the cell body. Note the presence of cytoplasmic microtubule(s) (absent in WT cells) at the proximal region of the basal body (asterisk). K, kinetoplast. Scale bar indicates 200 nm.

We used the tetracycline-inducible RNAi system to assess the function of BILBO1 in PF cells and BSF cells [29,30]. Cell growth was arrested in the PF cells after 24 h of induction, followed by cell death (as judged by a reduction in cell numbers over time) after 48–72 h of induction. In induced BSF cells, cell death, (as judged by a reduction in cell numbers over time), began after approximately 24 h of induction (Figure S1G and S1H). Note that western blot studies show that BILBO1 protein was not completely depleted in PF cells at 72 h after induction (Figure 2A). Densitometry data of PF cells indicate that at 24 h of induction, BILBO1 protein levels had dropped to 44.2% of parental levels and to 27.5% and 19.6% at 48 h and 72 h, respectively. When we observed PF cells by immunofluorescence after 36 h of BILBO1 RNAi induction, the BILBO1 signal was weak and only detectable on the mother FPC (unpublished data).

During BILBO1 RNAi induction, we observed that PF cells were elongated and supported new motile flagella but displayed an aberrant flagellum–cell body attachment. New flagella were attached to the cell body only through the basal body and were relocated to the distal portion of the aberrantly elongated posterior end of the cell. Antibody labelling of the basal body or paraflagellar rod (PFR) (a flagellar structure required for flagellar motility) indicated that the new flagellum was positive for the basal body and PFR proteins of these structures. The new flagellum was also closely associated with a new kinetoplast, as observed by immunofluorescence and DAPI staining (Figure 2B). Thin-section transmission electron microscopy observation of induced cells illustrated the astonishing finding that the FPs of these cells *were not* duplicated. Thus, no new FP were formed at the site of new flagellum growth (Figure 2D and 2E). However, the kinetoplast had duplicated and remained attached to, and was segregated by, the basal bodies of the new flagellum (Figure 2B, 2D, and 2E).

Taken together, these data indicate that (1) the formation of the FP requires BILBO1 protein and (2) that a reduction of BILBO1 protein levels by approximately 50% prevents FP formation and leads to cell death.

The image shown in Figure 2C illustrates a WT PF cell longitudinally sectioned at the FP level. This image clearly illustrates that the transition zone of the mature basal body (as shown by the arrowhead in the figure) is positioned within the FP lumen [31,32] and the PFR originates at the point where the axoneme exits the pocket [6,29]. In BILBO1 RNAi-induced cells, however, flagellum-to-cell body attachment has been disrupted, and the new basal body and transition zone are external to the cell body (Figure 2B and 2E). As with control cells, the origin of the new PFR in induced cells is also distal to the transition zone. Because the new flagellum of induced cells is attached to the cell only through the basal body region, this observation suggests that the axoneme itself contains the information necessary for determining where the PFR originates, as opposed to a signal or marker derived from attachment to the cell body. A higher magnification image of the basal body region of an induced cell (Figure 2E) illustrates the absence of a FP but also that the kinetoplast remains associated with, and segregated by, the basal body; it also shows the abnormal presence of microtubules in the cytoplasm at the proximal end of the basal body. The electron-dense material corresponding to the FPC at the exit site of the flagellum is clearly visible in noninduced cells but is not visible at the exit site of the new flagellum in induced cells, supporting the perception that BILBO1 RNAi cells do not form a new FP or a FPC (Figure 2D and 2E).

Overexpression of nontagged BILBO1 in PF cells did not produce any obvious aberrant phenotypes other than a slight delay in growth rate compared to WT cells (unpublished data). Immunofluorescence studies on cells overexpressing BILBO1 showed that the protein localised to both the mother and daughter FPCs (unpublished data). Intriguingly, overexpression of amino or carboxyl terminal eGFP-tagged BILBO1 for 24 h induced a large accumulation of the tagged protein at the mother FPC. Longer induction of eGFP-tagged BILBO1 (48 h) induced growth arrest (unpublished data). As with the RNAi cells, induction of eGFP-tagged BILBO1 produced cells with new motile flagella that were relocated to an aberrantly elongated posterior portion of the cell. These new flagella were not associated with any BILBO1 immunofluorescence signal or BILBO1-eGFP fluorescence signal, suggesting that no new FPC was formed. Induction of eGFP-tagged BILBO1 also initiated a disruption of new flagella-to-cell body attachment (unpublished data). Thin-section electron microscope images of these cells showed that they had accumulated abnormally large numbers of cytoplasmic vesicles, indicating that they had considerable endo- and exocytotic defects (unpublished data). These cells displayed similar phenotypes to the RNAi-induced cells described previously thus we propose that lethality is not due to overexpression of BILBO1-eGFP per se, but rather due to the inhibition of BILBO1 function via a dominant-negative effect.

**Figure 3:**
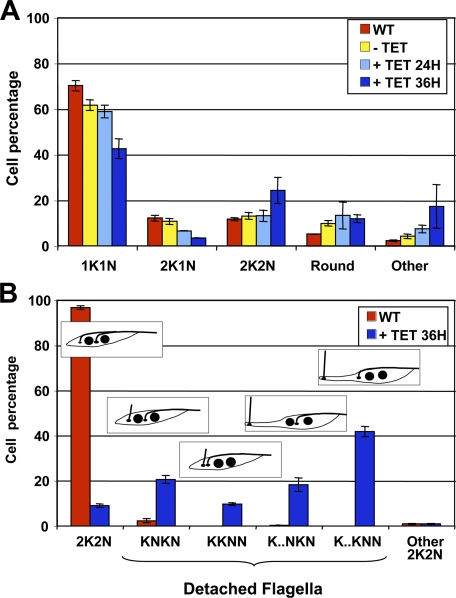
RNAi of BILBO1 Disrupts Cytokinesis (A) WT and BILBO1 RNAi PF cells were scored for kinetoplast/nuclei by DAPI labelling at 0, 24 h, and 36 h postinduction (+ TET). After 36 h of induction, a large number of 2K2N cell types are produced, and the proportion of the 2K1N cell type diminishes significantly. Induced cells arrest in the 2K2N configuration. The “Round” category represents round PF cells in which neither the nucleus nor the kinetoplasts could be individually distinguished. The “Other” categories represent cells in which 2N or 2K could not be assessed. (B) The distribution of 2K2N cell types in WT and BILBO1 RNAi-induced cells (36 h). Cell morphology was scored by phase contrast microscopy and DAPI labelling as well as for the number of cells with flagella that had lost their flagellum-to-cell body attachment. The “Other” 2K2N category represents PF cells in which the position of the flagellum could not be assessed. Five distinctive 2K2N phenotypes were observed in induced PF cells: (1) 2K2N cells that appeared normal in kinetoplast and nuclear positioning (KNKN [8.96% SE ± 0.82%]); (2) KNKN cells with a loss of new flagellum-to-cell body attachment (20.56% SE ± 1.76%); (3) KKNN cells with a loss of new flagellum-to-cell body attachment (9.63% SE ± 0.63%); (4) elongated KNKN cells (18.33% SE ± 3.01%); and (5) elongated KKNN cells (41.73% SE ± 2.3%).

Cell counts using DAPI-stained PF cells (Figure 3A) indicated that the ratio of cells with two kinetoplasts and two nuclei (2K2N) increased from 11.73% (standard error [SE] ± 0.63%, *n* = 1,542) in the nontransformed parental cell line to 24.41% (SE ± 5.73%, *n* = 811) in induced cells after 36 h of induction. We also detected a decrease in the population of cells with one kinetoplast and one nucleus (1K1N) from 70.24% (SE ± 2.22%, *n* = 1,542) in the parental cell line to 42.62% (SE ± 4.33%, *n* = 811) in induced cells after 36-h induction. Interestingly, only 3.57 ± 1.43% of the population were multinucleated, as compared to 1.46% ± 0.7 in the noninduced cells, indicating that induced cells do not continue through mitosis but instead undergo a cell-cycle block at the 2K2N stage (Figure 3A). Within the induced 2K2N population (Figure 3B), 60.06% (SE ± 2.76%, *n* = 535) of cells possessed an elongated posterior end. Furthermore, 91.04% of induced 2K2N cells had the mispositioned flagellar phenotype, 8.96% of noninduced cells had mispositioned flagella, whereas 3.36% of WT cells had this phenotype. In all BILBO1 RNAi-induced cells, mispositioned new flagella always maintained a disrupted flagellum-to-cell body attachment.

Five distinctive 2K2N phenotypes were observed in induced PF cells (Figure 3B): (1) 2K2N cells that appeared normal in kinetoplast and nuclear positioning (KNKN [8.96% SE ± 0.82%]), (2) KNKN cells with a disrupted loss of new flagellum–cell body attachment phenotype (20.56% SE ± 1.76%), (3) cells with two kinetoplasts and two nuclei positioned kinetoplast–kinetoplast, nucleus–nucleus (KKNN) with a disrupted loss of new flagellum–cell body attachment phenotype (9.63% SE ± 0.63%), (4) elongated KNKN cells (18.33% SE ± 3.01%), and (5) elongated KKNN cells (41.73% SE ± 2.3%) (Figure 3B). The reason for production of KKNN cells is not clear, but it may be related to where the cell is positioned within its cell cycle (e.g., early or late in mitosis) when new FPC biogenesis is inhibited by RNAi knockdown. Intriguingly, a KKNN organisation is observed in normal WT BSF trypanosomes, thus this organelle arrangement may reflect a modified mechanism of organelle segregation in BSF cells compared to PF cells. Noticeably, in all of the induced cells, the new flagella were shorter than the mother flagella (Figure 2B), suggesting that these cells were also experiencing difficulties in delivery of cargo for construction of the new flagellum.

**Figure 4:**
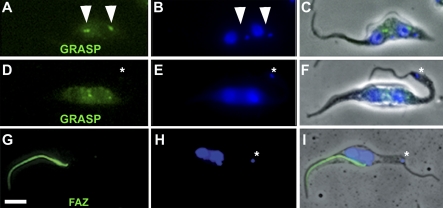
RNAi Knockdown of BILBO1 Induces Loss of Basal Body–Mediated Golgi Segregation and Causes Defects of Important Cytoskeletal Structures (A–C) A nontransformed 2K2N cell probed with anti-GRASP (green) and DAPI (blue), illustrating two major GRASP signals (arrowheads) located between the segregated kinetoplasts and nuclei. (D–F) A BILBO1 RNAi-induced (36 h) 2K2N cell probed with anti-GRASP. The two GRASP signals are observed near the nuclei. Despite a limited degree of Golgi segregation, no GRASP signal is observed near the new kinetoplast. The kinetoplast and the new flagellum (asterisk) are located in the extreme posterior end of the cell. (G–I) BILBO1 and the FPC are important for cytoskeleton organisation. Immunofluorescence micrograph of a PF cytoskeleton probed with L3B2 (anti-FAZ) antibody after BILBO1 RNAi knockdown (36 h). The flagellum-to-cell body attachment is lost, and the new flagellum is located at the posterior region of the cell. No new FAZ is formed, whereas the old FAZ remains associated with the old flagellum. The kinetoplast (asterisk) is located in the extreme posterior of the cell. Scale bar indicates 5 μm.

Electron microscopy reveals that induced PF cells possess what appear to be stacks of membranes that resemble a Golgi apparatus. These cells also amass large numbers of vesicles (Figure S1D and S1F). Since Golgi duplication in procyclic T. brucei cells involves Centrin-2 [33], and Golgi separation in T. brucei is basal body mediated [8], we wanted to test whether the observed Golgi swelling influenced Golgi duplication or segregation in induced BILBO1 RNAi-elongated cells. BILBO1-induced cells (36-h induction) were therefore probed with anti-GRASP antibody (Golgi marker) [33] and viewed by immunofluorescence to observe Golgi duplication and segregation. Similar to previous studies on WT cells, we observed two or more major separate Golgi-positive signals in all induced 2K2N phenotypes (Figure 4A–4F) [8,33]. The extended posterior portion of induced 2K2N cells varied in length; therefore, we scored Golgi signals of induced cells that were present in the extreme posterior distal half of the extension as “basal body segregation positive” and Golgi signals in the proximal anterior half as “basal body segregation negative.” In 2K2N WT cells, 98.56% (SE ± 0.26%, *n* = 764) were segregation positive, whereas 19.98% (SE ± 3.31%, *n* = 456) of induced cells (36-h induction) were segregation positive. These data indicate that in BILBO1 RNAi cells, Golgi duplication is not inhibited, but the basal body-dependent Golgi segregation machinery is disrupted. This latter observation is due, most likely, to malformations observed in the duplicated Golgi that may block the formation of essential components of the segregation machinery, but also probably related to the loss of cytoskeleton organisation and function in the absence of a FPC at the relocated posterior flagellum.

### RNAi of BILBO1 Inhibits the Formation of a Flagellum Attachment Zone Structure

In trypanosomes, a cytoskeletal structure, called the flagellum attachment zone (FAZ) is thought to be involved in the organisation of the flagellum and cytokinesis. This structure is located in the subpellicular cytoskeleton, where it subtends the flagellum [6,34,35]. FAZ proteins are required for flagellum attachment, and loss of the FAZ induces both a flagellum-to-cell body detachment and an inhibition of cytokinesis [36,37]. The L3B2 monoclonal antibody recognises the cytoplasmic filament of the FAZ in immunofluorescence and in immunoelectron microscopy [34]. We have used the anti-FAZ antibody L3B2 to study the organisation of the FAZ in the context of flagellum positioning in induced cells. Immunofluorescence studies demonstrate that in BILBO1 RNAi-induced PF cells, no new L3B2-positive FAZ filaments are formed, and the FAZ signal observed remains associated only with the old maternal flagellum (Figure 4G–4I). These data illustrate that flagellum and basal body formation are not sufficient for FAZ formation and could imply that the FPC or FP is required for FAZ formation. If this is the case, the absence of the FAZ could induce the absence of normal flagellum-to-cell body attachment. The lack of a FAZ has previously been observed to produce loss of flagellum-to-cell body attachment [36–39]. Alternatively, the absence of FAZ formation could be explained by the fact that new flagella exhibit a rapid flagellum-to-cell body detachment.

As the new flagellum of induced cells consistently exhibits a flagellum-to-cell body detachment, we therefore wanted to determine when in the cell cycle does flagellum detachment occur, and does the new flagellum remain associated to the old flagellum while within the FP? We probed PF cytoskeletons with AB1, which is a monoclonal antibody that localizes to a protein component of the flagellar connector (FC) [40]. The FC is a flagellum–flagellum linkage that is formed during cell division in PF cells [39]. It is present on the distal tip of the new flagellum and is normally tethered at the tip to the lateral aspect of the old flagellum. It is involved in the replication of the helical cell pattern and polarity of trypanosomes. Studies using trypanosome intraflagellar transport (IFT) knockdown cells showed that in the absence of a new flagellum, the FC can still migrate along the old flagellum. Therefore, new flagellum–FC attachment is not essential for FC movement and suggests that the FC has a novel motor for movement along the old flagellum [41].

**Figure 5:**
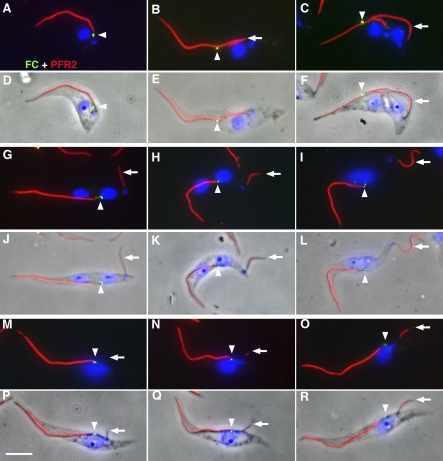
The New Flagellum of BILBO1 RNAi-Induced Cells Loses Physical Contact with the Old Flagellum Early in the Cell Cycle (A–F) Uninduced BILBO1 RNAi cytoskeletons probed with DAPI and double-labelled with anti-Flagellum Connector AB1 (green) and anti-PFR2 L8C4 (red) antibodies showing the attachment of the new flagellum to the maternal old flagellum and movement of the FC with the growth of the new flagellum. (A and D) Merged images of a 1K1N cytoskeleton in which the FC is located in the FP of the old flagellum. (B and E) Merged images of a 2K1N2F (two kinetoplasts, one nucleus, and two flagella) cytoskeleton in which the FC is present at the distal end of the new flagellum. (C and F) Merged images of a 2K2N2F postmitotic cytoskeleton in which the FC is present at the distal end of the new flagellum. (D, E, and F) are phase contrast merged images of (A, B, and C), respectively. (G–L) 2K2N2F-induced BILBO1 RNAi cytoskeletons showing the extended posterior end of the cell and the new flagellum-to-cell body attachment is disrupted. The FC is present only in the FP of the old flagellum. (J, K, and L) are phase contrast merged images of (G, H, and I), respectively. (M–R) 2K1N2F BILBO1 RNAi-induced cytoskeletons showing early stages in the phenotype in which the new flagellum is clearly not attached to the old flagellum. The FC is present only in the FP of the old flagellum. In (M and P), no PFR2 signal is detectable on the new flagellum. This new flagellum is at a very early stage of growth; it is not attached to the old flagellum and has a detached flagellum-to-cell body phenotype. The FC remains in the FP. (P, Q, and R) are phase contrast merges of (M, N, and O) respectively. Arrowheads denote the FC signal, and arrows denote the new flagellum. Scale bar indicates 5 μm.

AB1 labelling of noninduced cells showed the normal attachment of the new flagellum to the old flagellum within the FP, a similar observation to those published previously [40]. The immunofluorescence data presented in Figure 5A show a noninduced PF cell that has been probed with AB1 and a monoclonal antibody (L8C4) that targets the PFR. The merged immunofluorescence and phase contrast images of this cell indicate that a short new flagellum has formed, and the AB1 anti-FC staining shows the presence of the FC new-to-old flagellar attachment site. However, no L8C4 signal is observed on this new flagellum, indicating that it is only a few microns long and is located within the FP. As cells progress through the cell cycle (Figure 5B, 5C, 5E, and 5F), the new flagellum emerges from the FP. The distal tip of the new flagellum remains attached to, and moves along, the old flagellum (Figure 5A–5C).

BILBO1 RNAi-induced and AB1-probed cells showed that there was a FC-positive signal present on the old flagellum, but this signal remained in the FP (proximal to the origin of the old PFR signal) (Figure 5G–5L). Induced non-elongated cells also had formed the FC-positive signal, which, similar to elongated cells, remained in the FP. Based on its short length, and in comparison to WT cells, the new flagellum of an induced cell early in the cell cycle should normally be located within the FP. The example shown in Figure 5M and 5P illustrates that the new flagellum is PFR negative, indicating that it would normally be located within the FP and should be attached to the old flagellum. However, in this case, attachment of the new flagellum did not occur or was transient. The location of this short new flagellum indicates that the new basal body of BILBO1 RNAi-induced cells can “dock” in the proximity of the old pocket in a similar manner to that found in WT cells. Figure 5N and 5Q or Figure 5O and 5R illustrate induced cells with short flagella, but in both cases, they are PFR positive. In all induced cells, however, the new flagellum is never attached to the old flagellum even though the FC has formed. This suggests that attachment did not occur or was not stable enough to maintain the new-to-old flagellar linkage. If FC attachment did occur, it was lost at an early point in the growth stage of the new flagellum.

### Induced BILBO1 RNAi Cells Show No Endocytotic Activity Associated with the New Flagellum

**Figure 6:**
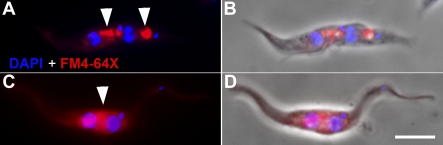
No Endocytotic Activity Is Associated with the New Flagellum A 2K2N WT cell (A and B) and a BILBO1 RNAi-induced cell for 36 h (C and D) were labelled with DAPI (blue) and the red fluorescent lipophilic dye FM4-64X (red). Arrowheads in (A) denote areas of endocytotic activity associated with both FPs of this cell. In (C), a large area of endocytotic activity, in the region of the old FP, is labelled, but no activity is associated with the new flagellum at the posterior end of the cell. (B and D) are DAPI-phase-fluorescence merged images of (A and C). Scale bar indicates 5 μm.

To test whether induced cells were capable of orthodox endocytotic activity, we carried out live PF cell endocytosis analysis using the fixable fluorescent lipophylic dye FM4-64X. WT 2K2N cells showed strong endocytotic activity at the base of both flagella, suggesting endocytosis activity via the old and the new FP (Figure 6A and 6B). In induced cells, we observed no endocytotic activity at the site of the new flagellum but considerable activity at the old FP (Figure 6C and 6D). Additionally, induced cells at the site of the new flagellum were negative for markers of early endocytosis such as clathrin or Rab5A (Figure S2), illustrating that in the absence of the FPC, and despite the fact that the new flagellum is still formed, no endocytotic activity is associated with this new flagellum.

In order to identify perturbations in trafficking and pocket targeting systems, we probed induced cells with (1) antiserum to the cysteine-rich acidic transmembrane protein (CRAM) (a FP protein of unknown function but postulated to be a lipoprotein receptor) [42]; (2) antiserum to procyclin, a major surface coat protein (GPEET) expressed in PF cells [43]; and (3) antiserum to p67, a lysosomal protein [44]. In all cases, noninduced cells gave localisation signals similar to control cells in work published previously. However, induced cells gave strong vesicle and/or vacuolar labelling patterns (Figures S3 and S4), and in the case of CRAM, the whole cytoplasm was positive for this protein (Figure S3E–S3H). CRAM localization was also checked by immunoelectron microscopy and confirmed that induced cells rapidly accumulate CRAM-positive vesicular structures (Figure S1E). Western blotting of BILBO1, clathrin, Rab5A, CRAM, and procyclin (GPEET) proteins after BILBO1 RNAi indicated that clathrin and Rab5A levels appear relatively constant, but that CRAM protein levels increased considerably (unpublished data).

Preliminary studies on cultured BSF cells show that there is a significant difference between the phenotype seen in PF cells versus that seen in BSF cells following BILBO1 ablation. In BSF cells, the immediate morphological effect observed was the rapid formation of spherical cells. Cells began rounding up as early as 12 h after RNAi induction. No aspects of the BSF cells were elongated after BILBO1 knockdown. This rounding up of induced cells prevented a clear analysis of kinetoplast and nucleus number or organisation (Figure S5). Furthermore, immunofluorescence labelling with the anti-PFR monoclonal antibody L8C4 showed that induced cells did not have the flagellum-to-cell body detachment phenotype observed in PF cells, and immunofluorescence labelling with the anti-FAZ monoclonal antibody L3B2 showed that in contrast to PF cells, induced cells often possessed two FAZ signals (unpublished data). Together, this indicates that BILBO1 RNAi in BSF cells has very different effects on cytoskeleton function and organisation in comparison to PF cells.

## Discussion

### BILBO1 Is a Component of the Flagellar Pocket Collar and Is Essential

We have identified a novel protein (BILBO1) that is located around the axoneme of T. brucei as it exits the FP. Using a variety of techniques, we have demonstrated that BILBO1 is part of a “horseshoe” or “ring” of a detergent-insoluble cytoskeletal structure known as the flagellar pocket collar (FPC). BILBO1 is the first component of the FPC to be identified and characterised. The FPC is important for the cell because it forms an “adhesion zone” of electron-dense material located between the pellicular, flagellar, and sequestered FP membranes [6,18]. New flagellum growth is supported in the absence of new FP construction in the case of RNAi knockdown of BILBO1, but the deficiency of a new FPC and FP directly or indirectly results in cell death in both insect and bloodstream forms of *T. brucei.*

In T. brucei, it has been demonstrated that FP selectivity exists to retain certain proteins since, for example, the CRAM protein and transferrin receptors are restricted to the FP, whereas procyclin and VSGs are found on the flagellum, FP, and cell surface [14,45]. The nature of this selectivity of distribution is unknown, but it may be developmentally regulated or associated with interactions between the FP membrane, the pellicular membrane, and the FPC.

Numerous structures that physically link axonemes to the cell body or the cytoskeleton have been identified in lower and higher eukaryotes [46–49]. One interesting example is the “ciliary necklace.” This structure has been identified in all 9+2 and 9+0 mammalian and invertebrate cilia, but it is not universally found in sperm. The necklace is located at the basal plate of cilia where the axonemal membrane “pinches in” [50]. Additionally, numerous proteins, including centrin, have been identified to be associated with basal bodies/centrioles and the cytoskeleton [51]. However, the molecular nature or function(s) of many of these structures remain to be identified [52]. The ciliary necklace-like structure of T. brucei is visible at the transition zone region, between the axoneme and the flagellar membrane [53], but it does not appear to be associated with the FPC [54]. Furthermore, extensive searches to define necklace proteins in any organism and to characterise their function have been fruitless. A more comprehensive search for these proteins should now be possible since centriole, cilia, and flagella proteomes have been published [28,55–58].

Recent evidence illustrates that certain primary cilia can function as sensory organelles that detect changes in fluid flow and initiate gene expression accordingly [52,59,60]. Notably, some of these primary cilia have structures similar to FPs with some electron-dense material at the exit point of the cilium, similar to the organisation of the FPC. The pocket-like structure is called the axonemal “vesicle” or sheath; it is thought to be Golgi derived and extends along with the growing ciliary axoneme within the cytoplasm [61]. The function of this vesicle is in all probability to provide a distinct, isolated compartment separated from the cytoplasm to allow intraflagellar transport for axonemal elongation. However, the molecular functions of the vesicle of primary cilia are not known.

Certain primary cilia can retract if subjected to physiological stress [62]. Membrane that is bound to the proximal region of the primary cilia axoneme is observed when they retract [63]. Presumably, the membrane in these structures is derived from the pellicular membrane, but exactly how this membrane is maintained as a uniform and organised “sack” or “pocket” around the primary cilia remains unresolved. Electron microscopy data suggest that the primary cilia vesicle is also able to carry out endocytotic activity via coated pits [64,65]. The presence of pits implies an organisation of membrane and proteins that separate the plasma membrane from the primary cilia vesicle. In this regard, we propose that a structure additional to the necklace and analogous to the FPC of trypanosomes may exist in primary cilia and may be important for positioning of cilia and, possibly, in trafficking processes.

In mammals and yeasts, actin and actin-binding proteins are the major cytoskeleton components associated with endo- and exocytosis [66,67]. These proteins are essential for the reshaping of the plasma membrane to facilitate endocytosis. They are often found associated with coated pits in the form of transient patches tightly associated with primary endocytotic vesicles. The actin poisons Latrunculin A and Jasplakinolide partially inhibit endocytosis in mammalian cells but initiate a complete endocytotic block in Saccharomyces cerevisiae [66–69]. In trypanosomes, actin has a differential role whereby it is essential and required for the formation and trafficking of endocytotic vesicles in BSF cells of T. brucei. Loss of actin by RNAi in BSF cells prevents endocytosis and results in enlargement of the FP, followed by cell death. In contrast, actin is neither essential nor associated with the FP in procyclic cells [4]. Furthermore, in trypanosomes, actin has not been observed as polymers or bundles, rather it localises to the endocytotic pathway but does not associate with the subpellicular cytoskeleton or the FP, illustrating that it is not a component of the FPC [4].

### Flagellar Pocket Collar Control Over Cell-Cycle Progression

The FAZ is thought to attach the trypanosome flagellum along the cell body and to coordinate correct cytokinesis [10]; thus, the FAZ plays an important role in the regulation of cell division. In BILBO1 RNAi-induced PF cells, the flagellum-to-cell body attachment of the new flagellum was disrupted and the expected new FAZ was absent, implicating an important relationship between the FAZ and the FPC/FP. The lack of FAZ formation is striking, but consistent with the orientation of the new flagellum being detached from the cell body. Alternatively, the FAZ is absent because the new flagellum (1) rapidly loses a flagellum–cell body attachment or (2) never initiates an attachment to the cell body. In either case, the absence of a new FAZ raises interesting questions regarding the control of FAZ formation and its relationship with other structures of the cytoskeleton.

A unique feature of the trypanosome cell cycle is that defects in cytokinesis do not necessarily trigger mitosis checkpoints, so that cells become multinucleated when cytokinesis is blocked [70]. However, in BILBO1 RNAi-induced cells, only 3.57% were multinucleated, suggesting the stimulation of a true cell-cycle block. This apparent S phase and mitotic block is unlikely to be due completely to the loss of FAZ. Previous studies have shown that interfering with correct FAZ formation, by RNAi knockdown of a FAZ protein called FLA1, induces flagellar detachment and cytokinesis block, but not mitosis, because induced FLA1 RNAi cells develop a multinucleated phenotype [36].

In BILBO1 RNAi-induced PF cells, the presence of a single FAZ may pose difficult cytokinesis-related problems for the cells. Our studies suggest that the FP or FPC plays a more substantial role in the cell cycle than does the FAZ; however, we are unable to define whether it is the FPC or the FP that initiates this cell cycle block. Even though BILBO1 is expressed in both PF and BSF cells, reduction of expression in PF cells induces the formation of many 2K2N cells that arrest in a phenotype in which basal bodies are located on the posterior side of the two nuclei (KKNN) instead of an alternated KNKN conformation. These data raise questions as to whether BILBO1 or the FPC, in interaction with the cytoskeleton, function in controlling cell-shape differentiation.

### The New Flagellum Does Not Require a Flagellar Pocket, Flagellar Pocket Collar, or Flagellar Connector to Grow

It appears that the new basal body of BILBO1 RNAi-induced cells can “dock” in the proximity of the old pocket in a fashion similar to that found in WT cells, but it then moves away to the extreme posterior end of the elongated cell, possibly because of failure to assemble the FP and FPC. It does not appear to depart from its position next to the old basal body, or migrate into the cytoplasm to an incorrect position, before extending a measurable length of axoneme. The mispositioning of the new flagellum towards the cell posterior and the absence or early loss of flagellum-to-flagellum attachment supports the supposition that the new flagellum grows into the pellicular membrane and/or remnants of the old FP membrane during or early after new axoneme growth is initiated.

In these induced cells, the PFR grows in parallel with the new flagellum and is independent of attachment to the cell body, thus indicating that the PFR is dependent on the axoneme for initiation and formation rather than signals from the cell body. Our data also show that new flagellar growth of procyclic cells is autonomous of the FC, or an attachment to the old flagellum. Indeed, new flagella of induced cells rapidly lose or may not establish flagellum-to-flagellum or flagellum-to-cell body attachment early in the cell cycle. The work of Davidge et al. (2006) [41] showed that new flagellum formation is not essential for FC movement. They also showed that a new FAZ was formed after intraflagellar transport (IFT) knockdown inhibited new flagellum growth. Why the FC is limited to the FP after BILBO1 RNAi remains to be determined, but it could be argued that absence of FC movement could be related to the absence of FAZ formation. To date, no proteins of the FC have been identified, thus the dependency relationships between the old and new flagella via the FC cannot as yet be studied in more detail.

The extreme posterior localisation of the new flagellum in BILBO1 RNAi-induced cells is intriguing and is observed only in 2K2N cells. This location in the cell cycle is not coincidental; otherwise, one would expect to observe the site of the new flagellum to be distributed randomly on the cell surface and in any cell-cycle stage. One possibility is that the new flagellum may be pushed to the posterior end of the cell by the growth of new or preexisting subpellicular microtubules. This would suggest a loss of control over the polymerisation of the microtubules involved in the posterior extension of the cell during normal division. Why subpellicular microtubules of induced cells elongate to such an extent is also interesting and requires further investigation.

Other workers have observed a posterior-end extension in trypanosomes after expression or RNAi knockdown of proteins related to differentiation or control of the cell cycle. Overexpression in PF cells of TbZFP2, a zinc finger protein implicated in differentiation from BSF to PF cells, causes a “nozzle” phenotype (a posterior extension of the cytoskeleton) as well as the occurrence of multinucleated and multiflagellated cells [71]. The authors showed that the nozzle is a result, at the posterior end of the cell, of polarized extension of microtubules rather than interdigitating short microtubules. RNAi depletion in PF cells of the cyclin CYC2, an essential PHO80-like cyclin, and the cyclin-related kinases CRK1+CRK2 also induced a polarized extension of posterior-end microtubules [72]. In all these studies, the extended or nozzle phenotypes were only observed in 1K1N/2K1N cells (cells arrested in G1), as opposed to the postmitotic 2K2N cell-cycle stage observed in BILBO1 knockdown PF cells. The absence of an elongated posterior end in BSF BILBO1 RNAi cells is similar to the observations of Hammarton et al. (2004) [73] in that RNAi of CYC2 induces a nozzle phenotype in PF cells, but not in BSF cells. The reasons for the production of nozzle or extended posterior-end phenotypes are unclear; however, these results clearly indicate that cytoskeleton elongation is heavily influenced by cell-cycle and/or cell-differentiation checkpoints.

### Function of the Flagellar Pocket Collar

**Figure 7:**
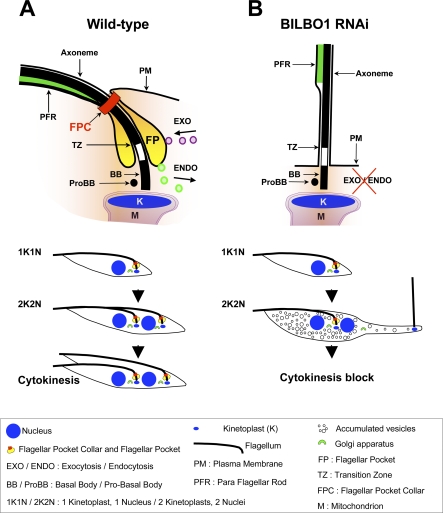
A Schematic Diagram That Describes the Normal Procyclic T. brucei Cell Division Cycle and the Fate of Induced BILBO1 RNAi Cells This schematic shows the organisation of the flagellum, function of the FP, and location of the FPC in a WT cell (A). The lower half of (A) illustrates the normal division cycle of the FP, Golgi, and flagellum in WT cells, whereas the drastically altered morphology of an induced BILBO1 RNAi cell (B) shows the absence of a FP and the loss of new flagellum-to-cell body attachment. The lower half of (B) shows an example of the major phenotype formed resulting from BILBO1 RNAi knockdown in procyclic cells. BILBO1 RNAi prevents FPC and FP biogenesis, disrupts endo-exocytosis, initiates cytokinesis block, and induces new flagellum cell body detachment and relocation to the posterior of the cell.

The FPC remains intact and attached to flagella after detergent and salt extraction. The FPC thus most likely consists of a complex of proteins in addition to BILBO1, because BILBO1 itself does not appear to have any obvious membrane-targeting domains, but it does have a large coiled-coil domain consistent with protein–protein interactions. One function of the FPC complex is to physically link the flagellum to the neck of the FP and the cell body. More precisely, this link would produce an intimate bridge between the FP membrane, pellicular membrane, and the flagellum membrane. This bridge complex forms a barrier or an adherens junction-like plaque between the flagellum and the subpellicular cytoskeleton. It is well documented that the trypanosome axoneme exits the FP via the FPC, but little data have been published on the organisation of this structure. It follows that structural homologs are likely to be present in many organisms in order to define and localise the exit site of cilia or axonemes. A schematic diagram of the positioning of the FPC and its role in noninduced or induced cells is shown in Figure 7. This figure also illustrates the distribution and organisation of organelles before and after BILBO1 RNAi knockdown.

BILBO1 RNAi-induced cells arrest and die before all BILBO1 protein is lost from the old FPC. The new flagellum of induced cells does not remain attached to the old flagellum via the FC, even within the old mature FP. Surprisingly, the membrane at the base of the new flagellum of induced cells is not capable of carrying out endocytotic function (as demonstrated using FM4-64FX uptake); however, we should consider the possibility that activity could be below the level of detection using this fluorescence-based assay. Nevertheless, with only one functional FP, BILBO1 RNAi-induced cells appear stressed in the sense that this single FP must function for two FPs; this probably induces an endocytotic imbalance. Loss of the FPC appears to disrupt all components of the endocytotic pathway as observed by electron microscopy or via endocytotic and lysosomal markers. Exocytosis is possibly disrupted also, which raises questions regarding the ability and consequences of these cell types to carry out procyclin, VSG, or invariant surface glycoprotein (ISG) trafficking in insect form or BSF cells.

In summary, FP biogenesis, endocytotic activity, flagellar positioning, and cell division all have strict dependency relationships with the FPC in PF trypanosomes. The discovery of BILBO1 and identification of its partners will facilitate studies on the trafficking of surface proteins involved in parasite survival strategies and on FP biogenesis. The identification of nonparasite-specific BILBO1 partner proteins may help to identify generic axoneme and cilia positioning structures.

## Materials and Methods

### Trypanosome strains.

Genomic DNA of Trypanosoma brucei TREU927/4 GUTat10.1 [74] was used to amplify by PCR the *BILBO1* ORF. T. brucei procyclic cell line EATRO1125-T7T and BSF line 427 90–13 single marker [75,76] were grown and transformed as described in [77]. Transformants were screened by immunofluorescence and cell morphology after tetracycline induction (1 μg · ml^−1^), and cloned.

### Flagella preparation electrophoresis.

Flagellar proteins were prepared as follows: 1 × 10^10^ T. brucei EATRO 1125 cells were harvested by centrifugation (1,000 × *g*, 20 °C, 10 min) washed in PBS (pH 7.2), 10 mM EDTA. Cells were lysed in PBS, 2 mM MgCl_2_, 0.25% NP40, and protease-inhibitors (539134; Calbiochem). Genomic DNA and total RNA were digested with 200 U Benzonase. Cytoskeletons were extracted in 1 M NaCl (final concentration) and incubated for 10 min on ice. Flagella were harvested (30 min, 4 °C, 8,422 × *g*), washed in PBS, 2 mM MgCl_2_, 36 U Benzonase, washed in PBS and stored in PBS at −80 °C.

### Separation and analysis of minor flagellar proteins, electrophoresis, and mass spectrometry.

Flagellar proteins (10 mg total) were preseparated in denaturing conditions (5% Ampholines [pH 3–10], 2% CHAPS, 7 M urea, 2 M thiourea, 50 mM DTT) on a Bio-Rad Rotofor. Individual fractions were run on SDS-PAGE gels, and protein bands (60 kDa to 80 kDa) were excised and subjected to liquid chromatography–tandem mass spectrometry (LC/MS/MS) analysis.

### Gene characterization.

The protein sequence of BILBO1 was identified using a signature of ten individual peptide sequences. The corresponding ORF was identified by WU-BLAST on GeneDB database has been deposited at GenBank.

### Plasmid constructs, RNAi, and overexpression.

The plasmid p3960SL contains a “sense/antisense” cassette targeting a 600-bp fragment of the *T. brucei BILBO1* ORF (nucleotide position 1 to 600) in the pLew100 vector [78] and was constructed as follows. A sense fragment of 600 bp was amplified by PCR (with Taq polymerase) using the primers HindIII-3960 (5′GGTCGCaagcttATGGCGTTTCTCGTACAAGTAGCA3′) and 3960–600-XbaI (5′CTCAACtctagaCACACGGTTACCCTTTACATCGA3′) and cloned into pCR2.1-TOPO (p39–600). A 650-bp antisense fragment was amplified with the primers BamHI-3960 and 3960–650-XbaI (5′GTAGCTtctagaAAGTTGAGATTAAACACAGTGAA3′) and cloned in the pCR2.1-TOPO (p39–650). After digestion of p3960–600 by HindIII-XbaI, and p3960–650 by BamHI-XbaI, the excised fragments were simultaneously cloned into pLew100 between the HindIII and BamHI sites (p3960SL). For RNAi in BSF, a fragment corresponding to the last 514 bp of *BILBO1* ORF was amplified by PCR (using the primers BamHI-514-3960 5′tcaggatccCAGAGACGCTGATATCGTGAAA3′ and 3960-HindIII 5′GGTCGCaagcttATGGCGTTTCTCGTACAAGTAGCA3′) cloned between the HindIII and BamHI sites of the p2T7–177 plasmid [79]. To overexpress in vivo the recombinant BILBO1-eGFP protein, the *BILBO1* ORF was amplified by PCR with the primers HindIII-3960 and 3960-NoStop-XbaI (5′ATATtctagaATCTCGCGGATAGGACCTC3′) and cloned into the pLew79-GFP1 vector [80] to make p3960-GFP.

### Antibody production.

We thank the following researchers for antibodies: K. Gull (anti-PFR2, L8C4, anti-FAZ, L3B2, anti-FC, AB1, and anti-basal body, BBA4), M. Field (anti-Rab5A and anti-Clathrin), G. Warren (anti-GRASP), M. Lee (anti-CRAM), J. Bangs (anti-p67), and I. Roditi (anti-GPEET procyclin). To produce anti-BILBO1 antiserum, two peptides (H2N-SFPSRPSISELTRSAE-CONH2 and H2N-GSRSPVSHRSESQQAR-CONH2) were synthesized, conjugated to carrier protein (Eurogentec), and then injected into rabbits to produce polyclonal antiserum. Additionally, recombinant 6 histidine-BILBO1 protein was overexpressed in bacteria, purified in urea on Ni-NTA resin, and then injected into a mouse which was then used to produce a monoclonal antibody. All antisera, including the monoclonal, were tested by immunofluorescence and western blotting.

### Western blotting.

Whole cells (1 × 10^7^ cells) or cytoskeleton proteins were prepared as described in [77]. Membranes were blocked 1 h in TBS, 0.2% Tween-20, 3% milk (or TBS, 5% milk for K1), incubated overnight at 4 °C with mouse polyclonal anti-BILBO1 antibody diluted in blocking buffer 1:200. After washing, anti-CRAM, anti-Clathrin, anti-RAB5A, or anti-GPEET procyclin (K1), diluted 1:2,000 was used as in [43]. Filters were processed as in [77] or [43]. Western blots were scanned at 300 dpi, and densitometry analysis was done using NIH Image 1.62.

### Immunofluorescence.

Whole cells were washed in PBS, then spread on poly- l-lysine–coated slides. For cytoskeleton preparations, cells were extracted with 0.25% NP40 in PIPES buffer (100 mM PIPES [pH 6.9], 1 mM MgCl_2_) for 5 min, and then washed twice in PIPES buffer. Cells or cytoskeletons were fixed in −20 °C methanol or 3.7% paraformaldehyde or 3.7% paraformaldehyde with 0.025% glutaraldehyde. In the latter case, cells were neutralised for 15 min in 200 mM glycine washed in PBS, blocked in 1%–10% bovine serum albumin (BSA), and probed with anti-FC (AB1) 1:5, anti-PFR2 (L8C4) neat, anti-basal bodies (BBA4) 1:20, anti-CRAM 1:250, anti-GRASP 1:300, anti-Clathrin 1:250, anti-RAB5A 1:250, anti-GPEET procyclin (K1) 1:200, anti-p67 1: 400, or anti-BILBO1 rabbit polyclonal 1:500 and anti-BILBO1 monoclonal 1:10. Slides were processed as in [77] using Jackson, Sigma, or Molecular Probes secondary anti-IgG– or anti-IgM–specific secondary antibodies conjugated to FITC, Oregon Green, or Texas Red. Prior to K1 labelling, cells were permeabilised in 0.1% NP40 in PBS for 1 min, and then washed 3 × 10 min in PBS. Prior to Clathrin, RAB5A, and CRAM labelling, cells were permeabilised in 0.1% Triton X-100 for 1 or 10 min and blocked with BSA (1% or 10%, respectively for 10 min to 1 h). Slides were DAPI stained and mounted with Slowfade Lite (Molecular Probes). Images were acquired with Metavue 4.4 software, on a Zeiss Axioplan 2 microscope, using a Roper CCD 1300-Y/S digital camera, and processed with Adobe Photoshop 8. Brightness was reduced on DAPI images when used in merged presentations.

### Endocytosis assay.

Microscopic analysis of FM4-64FX uptake was carried out by a modification of the assay described by Hall et al., 2005 [81]. A total of 1 × 10^7^ induced (36 h) and noninduced BILBO1 RNAi procyclic cultures were harvested by centrifugation, washed in PBS, and then resuspended in 1 ml of PBS, 0.1 mM adenosine, and 10 mM glucose. FM4-64FX was added (to a final concentration of 2.5 μg/ml), and the cells were incubated for 15 min in the dark with mild agitation (25 rpm) on a rotary shaker. After incubation, the cells were kept on ice to block endocytotic activity. All following solutions and protocols were done at 4 °C. The cells were washed in PBS, deposited on poly-l-lysine–coated slides for 5 min in the dark, and then fixed for 15 min in the dark with 4% paraformaldehyde in PBS, followed by two 5-min washes in PBS. Finally, the slide was DAPI stained, 10 μg/ml, in PBS for 4 min, washed 2 × 5 min in PBS, mounted, and then viewed at room temperature as for immunofluorescence.

### Electron microscopy.

A total of 50 ml of mid-log phase WT or 48-h RNAi-induced cells were harvested by centrifugation at 1,000 × *g* for 15 min. Block preparation and protocol was performed exactly as in [77].

### Immunoelectron microscopy.

A total of 50 ml of RNAi-induced cells (36 h) at 1 × 10^7^/ml were harvested and resuspended in 25 ml of 4% paraformaldehyde, 0.025% glutaraldehyde in PBS for 2 h. Fixed cells were washed, dehydrated, and embedded in Lowicryl HM20 mono-step (EMS). Sections were cut, neutralised in 100 mM glycine for 10 min, blocked in PBS 1% BSA for 10 min, and probed with anti-CRAM 1:250 in blocking buffer 4 °C overnight. Sections were washed 4 × 10 min in PBS 1% BSA, then probed with 10-nm gold-conjugated protein A or G (Aurion) 1:30 in blocking buffer for 2 h. Sections were washed 4 × 10 min in PBS 1% BSA, then 4 × 10 min PBS, fixed in 1% glutaraldehyde in water for 1 min, stained in 2% uranyl acetate for 15 min, washed 4 × 5 min in water, and then viewed as described in [77]. Cytoskeletons were prepared as above with the following exceptions: extraction was in 25 ml of 1% NP40 in Pipes buffer, washed in Pipes buffer, and resuspended in 25 ml of 4% paraformaldehyde, 0.25% glutaraldehyde in PBS for 30 min. Blocks were prepared as above and probed with mouse anti-BILBO1 polyclonal antiserum 1:50 at 4 °C overnight. Sections were washed, probed with 10-nm gold-conjugated anti-mouse 1:30 and processed as above.

### Statistics.

Standard error was used for cell counts and *n* = 3 (3 different experiments in all cases). Sample size (n) is indicated as total cell number counted for the three experiments.

## Supporting Information

Figure S1 Quantification of BILBO1-eGFP Overexpression (A) Western-blot of whole PF cell extracts expressing BILBO1-eGFP probed with anti-BILBO1 (upper panel) and anti-PFR2 as loading control (lower panel). The quantification by densitometry of the expression of total BILBO1 protein (corrected by the PFR2 loading control) shows more than a 5-fold increase at 24 h or 48 h of induction. The numbers between the panels indicate the percentage of expression where 100% is WT control expression level.
(B) BILBO1 is expressed in BSF. A BSF 2K2N cytoskeleton probed with anti-BILBO1 showing the two FPCs at the proximal end of the old and the new flagellum. Scale bar indicates 5μm.
(C) A comparative western blot of PF cytoskeletons and flagella probed with anti-BILBO1 and anti-PFR2. Quantification of BILBO1 and PFR2 by densitometry shows an equivalent reduction of BILBO1 and PFR2 protein levels during the extraction process, suggesting that BILBO1 is not specifically extracted. After flagella preparation and blotting, the PFR2 signal is reduced to 59.2% of the cytoskeleton signal. The reduction in the PFR2 signal after cytoskeleton extraction is a result of the loss of flagella during the purification step. The corresponding flagellum BILBO1 protein level diminishes to 61.5% of the cytoskeleton signal. Although, in general, there is some flagellar loss, BILBO1 protein remains associated with extracted flagella under high salt and detergent conditions. Ck, cytoskeleton; Fg, flagella.
(D) A thin-section electron micrograph of a BILBO1 RNAi cell after 36 h of induction. Numerous abnormal cytoplasmic vesicles are present (arrowheads), whereas the Golgi-like apparatus appears enlarged and deformed as seen in (F).
(E) An electron micrograph of a thin section of a procyclic BILBO1 RNAi-induced cell (36 h) probed with anti-CRAM antiserum. Intense immunolabelling is observed on vesicles (note; vesicles collapse into larger structures due to the fixation conditions required for immunoelectron microscopy). CRAM label is specific to these vesicles, and little or no signal is observed as background. Scale bar indicates 1 μm.
(F) A thin-section electron micrograph of a BILBO1 RNAi cell after 36 h of induction showing an enlarged and deformed Golgi like apparatus.
(G and H) Growth curves of BILBO1 RNAi cell lines. Not transformed (NT), noninduced (−TET), and induced (+TET) PF and BSF cells.
(6.05 MB PDF)

Figure S2 RNAi of BILBO1 Disrupts Endocytosis (A–D) A nontransformed PF cell probed with anti-clathrin antibody (green) and stained with DAPI (blue). Anti-clathrin + DAPI merged images illustrate a major (near the old FP) and a minor (near the new FP) clathrin signal (arrowheads). (D) Merged images of (A–C).
(E–H) After 36 h of BILBO1 RNAi induction, clathrin-coated vesicles (arrowhead) are in close proximity to the mother FP only. (H) Merge of (E–G).
(I–L) Nontransformed cell probed with anti-Rab5A (green) and DAPI (blue). Major Rab5A signals (arrowheads) are located between kinetoplasts and the nuclei. Additional foci of Rab5A labelling adjacent to the old pocket and the new nucleus can also be observed and represent early endocytotic vesicles. (L) A merged phase-contrast image of (I–K).
(M–P) After 36 h of RNAi, Rab5A signals (arrowhead) are in close proximity to the mother FP only. The new kinetoplast and flagellum are located in the posterior extended tip of the cell (asterisk). (P) Merged phase contrast image of (M–O).
Scale bar indicates 5 μm.
(2.28 MB PDF)

Figure S3 BILBO1 RNAi Knockdown Induces Accumulation of CRAM-Positive Vesicles (A–D) A nontransformed 2K2N PF cell probed with anti-CRAM (green) and counterstained with DAPI (B) (blue). (C) An anti-CRAM + DAPI merged image illustrating two major CRAM signals (arrowheads) located between the segregated kinetoplasts and nuclei. (D) A phase contrast-merged image of (A–C).
(E–H) After 36 h of BILBO1 RNAi induction, the CRAM signal is observed throughout the cytoplasm and colocalises with the abnormal vesicles present between both nuclei. The new kinetoplast and flagellum are located in the posterior extended tip of the cell (asterisk). (H) A phase contrast merged image of (E–G).
Scale bar = 5 μm.
(3.93 MB PDF)

Figure S4 BILBO1 RNAi Knockdown Induces Accumulation of Procyclin- and p67-Positive Vesicles (A and B) A noninduced 2K2N PF cell probed with anti-procyclin and counterstained with DAPI. A uniform green label is observed on the cell surface. (B) A phase contrast, DAPI-stained merged image of (A).
(C and D) After 36 h of BILBO1 RNAi induction, a major procyclin signal is observed that localises with one or numerous abnormal vesicles present between both nuclei (arrowhead). (D) A phase contrast, DAPI-stained merged image of (C).
(E and F) A noninduced 2K2N PF cell probed with the lysosomal marker anti-p67. A focused label (green) is observed between the kinetoplast and nuclei. (F) A phase contrast, DAPI-stained merged image of (E).
(G and H) After 36 h of BILBO1 RNAi induction, a major p67 signal is observed to colocalise with the abnormal vesicles present between the nuclei (arrowhead). (H) A phase contrast, DAPI-merged image of (G).
The new kinetoplast and flagellum are located in the posterior tip of the cell (asterisk) ([C and D] and [G and H]). Scale bar = 5 μm.
(10.84 MB PSD)

Figure S5 Morphological Features Induced by BILBO1 RNAi Knockdown in BSF Cells. (A and B) DAPI and DAPI/phase contrast micrographs of a field of noninduced BILBO1 RNAi culture form BSF cells.
(C and D) DAPI and DAPI/phase micrographs of a field of induced (12h) BILBO1 RNAi culture form BSF cells, showing that some cells are rounding up.
(E and F) DAPI and DAPI/phase micrographs of a field of induced (24 h) BILBO1 RNAi culture form BSF cells. After 24 h of BSF induction, most cells are rounded, and no aspect of the cells was elongated after BILBO1 knockdown.
Scale bar indicates 5 μm.
(7.73 MB PDF)

### Accession Numbers

The GenBank (http://www.ncbi.nlm.nih.gov/Genbank) accession number for *BILBO1* is DQ054527, and the GeneDB (http://www.genedb.org/) accession number is Tb11.01.3960.

## Abbreviations

1K1N: one kinetoplast and one nucleus
2K2N: two kinetoplasts and two nuclei
BSF: bloodstream form
CRAM: cysteine-rich acidic transmembrane protein
eGFP: enhanced green fluorescent protein
FAZ: flagellum attachment zone
FC: flagellar connector
FP: flagellar pocket
FPC: flagellar pocket collar
GPI: glycosylphosphatidylinositol
KKNN: two kinetoplasts and two nuclei positioned kinetoplast–kinetoplast, nucleus–nucleus
KNKN: two kinetoplasts and two nuclei positioned kinetoplast–nucleus, kinetoplast–nucleus
NT: not transformed, PF, procyclic form
PFR: paraflagellar rod
RNAi: RNA interference
SE: standard error
VSG: variable surface glycoprotein
WT: wild-type
