# Supplementary material for: Biogenesis of the Trypanosome Endo-Exocytotic Organelle Is Cytoskeleton Mediated
Source: PLoS Biol. 2008 May 6;6(5):e105. doi: 10.1371/journal.pbio.0060105 (PMC2365980; doi:10.1371/journal.pbio.0060105)
Supplement: Supplementary file 1 [file pbio.0060105.sg001.pdf]

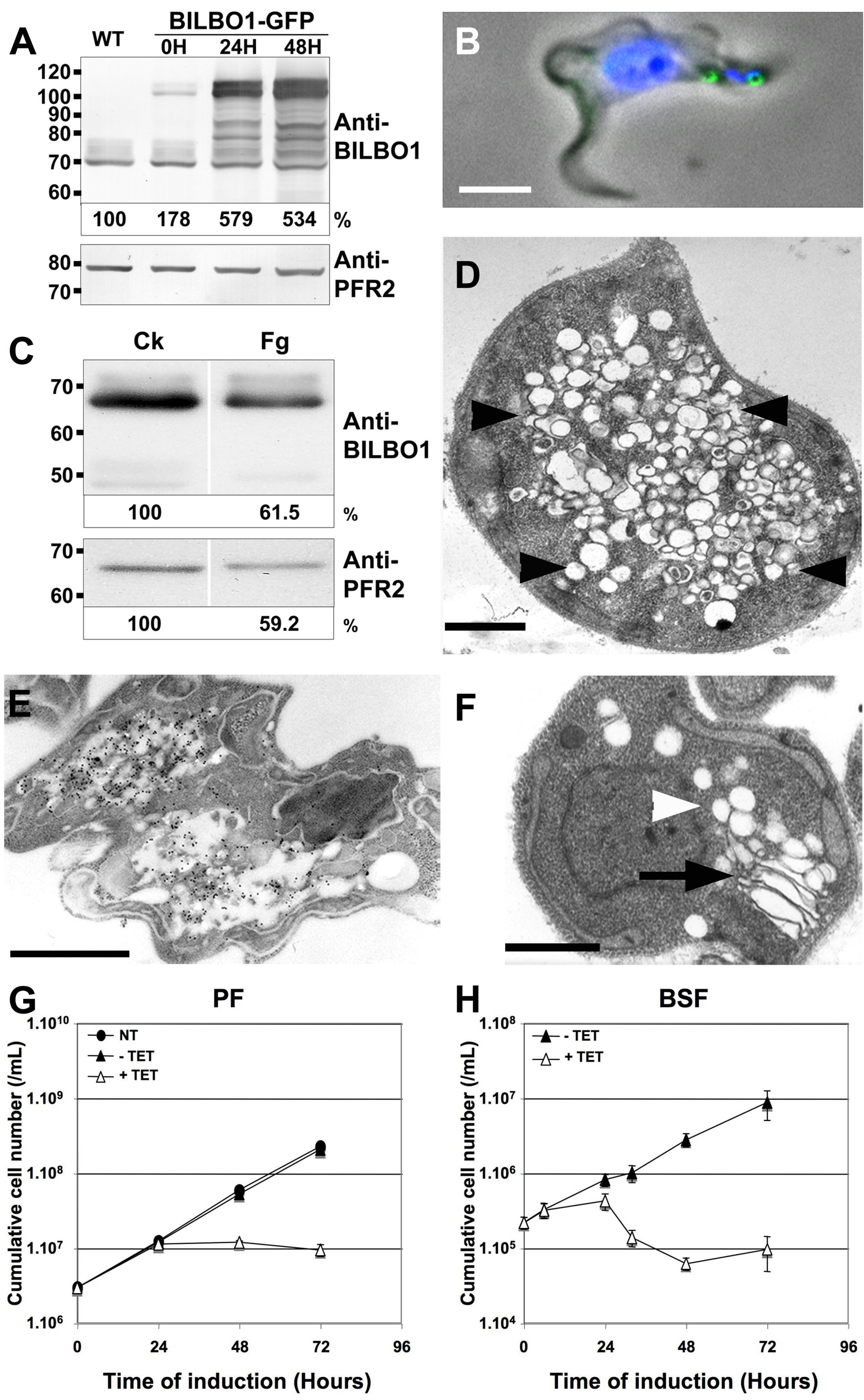

A
WT
BILBO1-GFP
0H
24H
48H
120
100
90
80
70
60
Anti-BILBO1
100
178
579
534
%
80
70
Anti-PFR2
B
C
Ck
Fg
70
60
50
Anti-BILBO1
100
61.5
%
70
60
Anti-PFR2
100
59.2
%
D
E
F
G
PF
NT
- TET
+ TET
Cumulative cell number (/mL)
1.10^{10}
1.10^{9}
1.10^{8}
1.10^{7}
1.10^{6}
0
24
48
72
96
Time of induction (Hours)
H
BSF
- TET
+ TET
Cumulative cell number (/mL)
1.10^{8}
1.10^{7}
1.10^{6}
1.10^{5}
1.10^{4}
0
24
48
72
96
Time of induction (Hours)
