# Supplementary material for: Biogenesis of the Trypanosome Endo-Exocytotic Organelle Is Cytoskeleton Mediated
Source: PLoS Biol. 2008 May 6;6(5):e105. doi: 10.1371/journal.pbio.0060105 (PMC2365980; doi:10.1371/journal.pbio.0060105)
Supplement: Supplementary file 3 [file pbio.0060105.sg003.pdf]

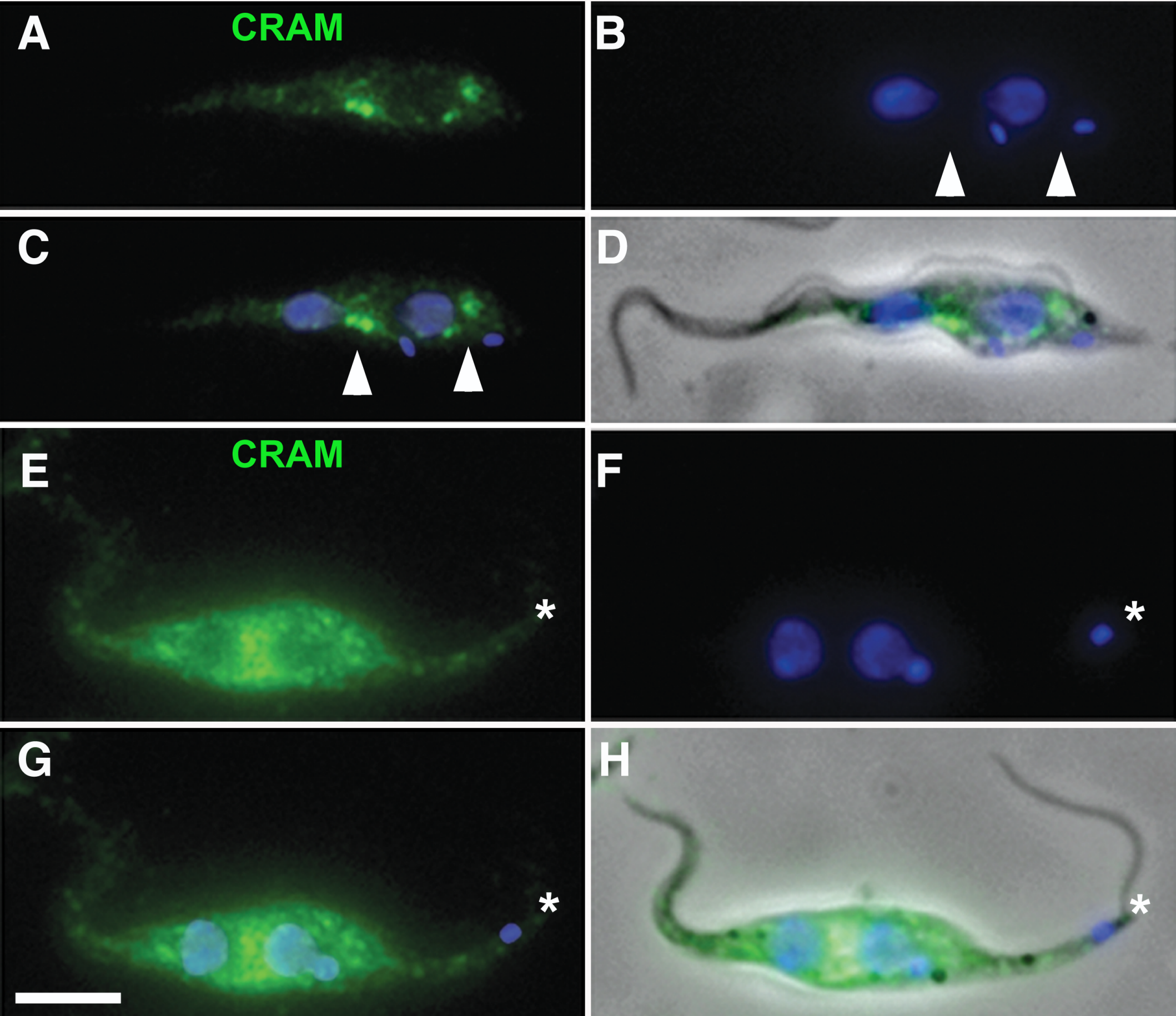
A
CRAM
B
C
D
E
CRAM
F
G
H
